# Unemployment, homelessness, and other societal outcomes in patients with schizophrenia: a real-world retrospective cohort study of the United States Veterans Health Administration database: Societal burden of schizophrenia among US veterans

**DOI:** 10.1186/s12888-022-04022-x

**Published:** 2022-07-08

**Authors:** Dee Lin, Hyunchung Kim, Keiko Wada, Maya Aboumrad, Ethan Powell, Gabrielle Zwain, Carmela Benson, Aimee M. Near

**Affiliations:** 1 Janssen Scientific Affairs, LLC, Titusville, NJ USA; 2 IQVIA, 4820 Emperor Blvd, Durham, NC 27703 USA; 3 Clinical Epidemiology Program, White River Junction VA Medical Center, White River Junction, VT USA

**Keywords:** Schizophrenia, Societal burden, Disease burden, Veterans, Homelessness, Unhoused, Unemployment, Premature mortality, Substance use disorder

## Abstract

**Background:**

The burden associated with schizophrenia is substantial. Impacts on the individual, healthcare system, and society may be particularly striking within the veteran population due to the presence of physical and mental health comorbidities. Disease burden is also influenced by a complex interplay between social determinants of health and health disparities. The objective of the current study was to compare non-healthcare societal outcomes between veterans with and without schizophrenia in the United States Veterans Health Administration (VHA).

**Methods:**

A retrospective cohort study was conducted using the VHA database (01/2013–09/2019; study period). Veterans with schizophrenia (≥2 diagnoses of ICD-9295.xx, ICD-10 F20.x, F21, and/or F25.x during the study period) were identified; the index date was the earliest observed schizophrenia diagnosis. Veterans with schizophrenia were propensity score-matched to those without schizophrenia using baseline characteristics. A 12-month baseline and variable follow-up period were applied. The frequency of unemployment, divorce, incarceration, premature death, and homelessness were compared between the matched cohorts using standardized mean difference (SMD). Risk of unemployment and homelessness were estimated using logistic regression models.

**Results:**

A total of 102,207 veterans remained in each cohort after matching (91% male; 61% White [per AMA]; median age, 59 years). Among veterans with schizophrenia, 42% had a substance use disorder and 30% had mental health-related comorbidities, compared with 25 and 15%, respectively, of veterans without schizophrenia. Veterans with schizophrenia were more likely to experience unemployment (69% vs. 41%; SMD: 0.81), divorce (35% vs. 28%; SMD: 0.67), homelessness (28% vs. 7%; SMD: 0.57), incarceration (0.4% vs. 0.1%; SMD: 0.47), and premature death (14% vs. 12%; SMD < 0.1) than veterans without schizophrenia. After further adjustments, the risk of unemployment and of homelessness were 5.4 and 4.5 times higher among veterans with versus without schizophrenia. Other predictors of unemployment included Black [per AMA] race and history of substance use disorder; for homelessness, younger age (18–34 years) and history of mental health-related comorbidities were additional predictors.

**Conclusion:**

A greater likelihood of adverse societal outcomes was observed among veterans with versus without schizophrenia. Given their elevated risk for unemployment and homelessness, veterans with schizophrenia should be a focus of targeted, multifactorial interventions to reduce disease burden.

## Background

Schizophrenia is a severe mental health disorder, affecting more than 23 million individuals globally, including approximately 3 million people in the United States (US) [1]. Schizophrenia increases the risk for premature mortality [2], resulting in an estimated average of 28.5 years of potential life lost [3]. Importantly, the prevalence of mental health disorders including schizophrenia is particularly high among US veterans [4], with the most recent estimates reporting 100,000–120,000 US veterans living with schizophrenia between 2010 and 2013 [2, 5, 6].

The economic burden associated with schizophrenia in the US has increased over the past decade [7]. The overall cost of schizophrenia, including direct healthcare costs (e.g., medications, hospitalization, outpatient and emergency department [ED] visits), direct non-healthcare costs (e.g., homeless shelters and incarceration), and indirect costs (e.g., lost productivity and premature mortality) was estimated to be $63 billion in 2002 [8]. By 2013, the overall cost was more than $155 billion, with indirect costs being the largest driver at $117 billion (76% of total costs) [9]. Similar trends were observed in several other studies, where the proportion of indirect costs ranged from 50 to 81% of the total economic burden associated with schizophrenia [8, 10–12]. Specifically, schizophrenia has been associated with higher unemployment rates (approximately 90% for working age patients with schizophrenia vs. < 10% for the general population) [3, 13, 14], and a higher prevalence of homelessness and incarceration, leading to increased demand for societal resources such as supported housing and rehabilitation services [15–17].

The burden of schizophrenia may be particularly pronounced in younger patients because they are at increased risk for poor treatment adherence and disease relapse [1, 18–20]. A recent meta-analysis of 41 global studies found that younger age of onset was associated with an increased number of relapses and poorer social and occupational functioning [20]. Another study estimating the economic burden among commercially insured patients with schizophrenia reported that young adult patients (aged 18–35 years) with schizophrenia had more inpatient stays (6.8 vs. 4.0) per year and higher inpatient costs ($15,692 vs. $10,274) than older patients [21]. Higher risk of suicide completion among younger individuals with schizophrenia has been also reported [22].

While the substantial burden of schizophrenia has been well-documented in the general US population, to the best of our knowledge, there is no literature describing societal outcomes within the US Veterans Health Administration (VHA) population, among whom the prevalence of mental health disorders, including schizophrenia, is high relative to the general population [4]. Therefore, we conducted a retrospective study to fill this research gap by describing and comparing the frequency of non-healthcare societal outcomes among veterans with and without schizophrenia in the VHA. With increasing risk of mental health illnesses and associated cost in the VHA system observed in recent years [23], this study will provide valuable insights on the disease burden among veterans with schizophrenia.

## Methods

### Data source

VHA is the largest integrated healthcare system in the US, providing health services to more than 9 million veterans. Clinical and administrative data were extracted from the integrated databases of the VHA Corporate Data Warehouse. This included VHA’s electronic medical records, which capture information from inpatient, outpatient, and ED visits for all persons treated within the VHA, as well as the VHA Vital Status files, which contain demographic and mortality information obtained from multiple VHA and non-VHA sources (including Medicare Vital Status and the National Death Index [NDI]).

All study procedures were carried out in compliance with federal and institutional ethical guidelines. The Veterans Affairs Central Institutional Review Board (IRBnetID: 1478231–1) provided approval for this study on December 17, 2019. The requirement to obtain informed consent from study participants was waived because the Institutional Review Board deemed this study to involve no more than a minimal risk to the privacy of individuals. Data used in the study were de-identified, and only aggregated results were shared.

### Study design

**Fig. 1:**
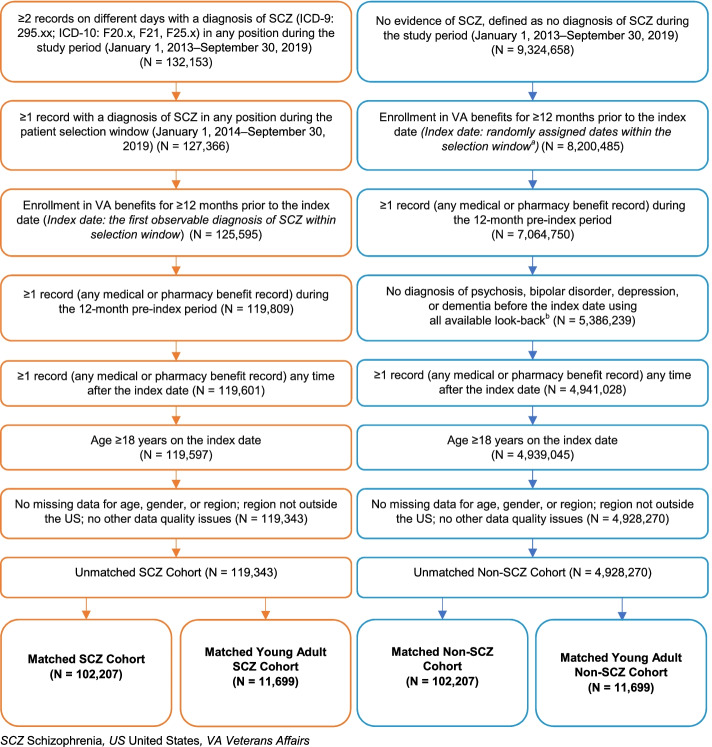
Patient selection criteria and attrition. a) The distribution of index year for the SCZ cohort informed the distribution of index year for the non-SCZ cohort at the time of matching. b) Psychosis, bipolar disorder, depression, manic depression, or dementia were identified with ICD diagnosis codes; a full list of codes can be provided upon request

We conducted a retrospective matched cohort study using the US VHA data from January 1, 2013 to September 30, 2019 (study period). Two cohorts of veterans were identified: the schizophrenia cohort and the non-schizophrenia cohort. Both cohorts included veterans aged 18 years and older who were enrolled in VA benefits for at least 12 months (see Fig. 1 for inclusion and exclusion criteria). The index date for the schizophrenia cohort was defined as the first observable diagnosis of schizophrenia between January 1, 2014, and September 30, 2019 (selection window). Veterans were followed until disenrollment from VA benefits, end of the data period, death, or end of continuous enrollment, whichever occurred first. In addition, a subset of the schizophrenia cohort, consisting of young veterans (aged 18–35 years), was created to represent veterans with schizophrenia who were likely to be at an early stage of the disease [21].

### Baseline patient characteristics

Baseline socio-demographic (age, sex, geographic region, race, urban vs. rural residence) and clinical characteristics, including history of substance use disorder (identified by ≥1 record of ICD-10 codes F10.x–F19.x and/or corresponding ICD-9 codes [303.x–305.x]), Charlson Comorbidity Index (CCI), comorbidities of interest (e.g., mental health-related comorbidities identified by corresponding diagnosis codes), and self-reported Patient Health Questionnaire-2 (PHQ-2) scores were assessed during the 12-month pre-index period for each cohort. The PHQ-2 contains questions about the frequency of depressed mood and anhedonia over the past 2 weeks; scores range from 0 to 6, with a score of 3 or higher considered the optimal cut-point when using the PHQ-2 to screen for depression (i.e., if the score is 3 or greater, major depressive disorder is likely) [24]. Diagnostic codes for other conditions can be provided upon request.

### Outcomes

Mortality outcomes included death, age at death, and frequency and causes of premature death during the variable follow-up period. Premature death was defined as death < 80 years old based on the definition used by the Centers for Disease Control and Prevention [25]. Cause of death was reported only for the schizophrenia cohort, using the available NDI data for the specific cause of death and an ICD-10 lookup table for the high-level classification of cause of death, supplemented by manual classification of certain key conditions. Non-healthcare societal burden measures included unemployment, divorce, homelessness, and incarceration and were reported using the most recently observed measure during the study period.

### Statistical analysis

Veterans in the schizophrenia cohort were 1:1 propensity score matched to veterans in the non-schizophrenia cohort to ensure the balance of baseline demographic and clinical characteristics; greedy nearest neighbor matching without replacement (caliper of 0.01) was used. Given the large sample size for the non-schizophrenia cohort, a representative sample of 500,000 veterans without schizophrenia meeting the inclusion and exclusion criteria were considered for matching. The propensity score model included age, sex, race, geographic region of residence, urban vs. rural residence, index year, and CCI. The matched cohorts were considered well-balanced for a given variable if the absolute standardized mean difference (SMD) between the cohorts was 0.1 or less. Additionally, young veterans (aged 18–35 years) with schizophrenia were similarly matched to young veterans from the non-schizophrenia cohort.

All study measures were summarized using descriptive statistics. For descriptive analyses, categorical measures were reported as frequency and percentage, and continuous variables were described as mean, standard deviation (SD), first quartile, median, third quartile, minimum, and maximum. The matched cohorts were compared using SMD, with any value of 0.1 or greater considered statistically meaningful. Data were analyzed using SAS version 9.4 (SAS Institute Inc., Cary, North Carolina).

The risks of unemployment and homelessness were compared between the schizophrenia and non-schizophrenia cohorts using logistic regression models, adjusting for age group, sex, race, geographic region of residence, mental health-related comorbidities, substance use disorder, and CCI. Only 2 of the 4 non-healthcare societal burden measures (i.e., unemployment and homelessness) were further adjusted for these covariates because of the SMDs observed post-matching and because improvements in unemployment and homelessness have been associated with positive effects on health outcomes for patients with schizophrenia [26–28]. Odds ratios (ORs) and 95% confidence internals (CIs) were reported.

## Results

### Baseline demographic and clinical characteristics

After matching, 102,207 veterans remained in each of the cohorts (Fig. 1). The overall study population was 91% male and 61% White and had a median age of 59 years.

The matched cohorts were generally well-balanced (Table 1). Imbalances between the 2 cohorts were observed for history of substance use disorder (42.2% for the schizophrenia cohort vs. 24.9% for the non-schizophrenia cohort; SMD: 0.37), mental health-related comorbidities (29.5% vs. 14.7%; SMD: 0.36), and PHQ-2 scores (mean 1.1 vs. 0.5; SMD: 0.41).

**Table 1:** Demographic and baseline clinical characteristics of the matched populations

	SCZ Cohort (***N*** = 102,207)	Non-SCZ Cohort (***N*** = 102,207)	SMD	Young Adult SCZ Cohort (***N*** = 11,699)	Young Adult Non-SCZ Cohort (***N*** = 11,699)	SMD
**Age at Index (years)**						
Mean (SD)	56.7 (13.8)	57.7 (16.3)	−0.06	29.7 (3.7)	30.7 (4.2)	−0.25
Median (IQR)	59 (50–66)	59 (47–67)		30 (27–33)	31 (28–33)	
**Age Categories,** *** n*** **, (%)**			0.10			0.00
18–34	10,205 (10.0%)	11,641 (11.4%)		10,613 (90.8%)	10,610 (90.7%)	
35–44	8774 (8.6%)	11,314 (11.1%)		1086 (9.3%)	1089 (9.3%)^b^	
45–54	16,798 (16.4%)	16,022 (15.7%)		NA	NA	
55–64	35,154 (34.4%)	31,908 (31.2%)		NA	NA	
≥ 65	31,276 (30.6%)	31,322 (30.6%)		NA	NA	
**Male Gender,** *** n*** **, (%)**	93,100 (91.1%)	92,469 (90.5%)	0.02	10,182 (87.0%)	10,202 (87.2%)	−0.01
**Race,** *** n*** **, (%)**			0.08			0.11
White	62,724 (61.4%)	62,889 (61.5%)		6801 (58.1%)	6782 (58.0%)	
Black	29,562 (28.9%)	28,979 (28.4%)		3513 (30.1%)	3411 (29.2%)	
Hawaiian Pacific islander	1233 (1.2%)	1187 (1.2%)		172 (1.5%)	180 (1.5%)	
Asian	886 (0.9%)	1238 (1.2%)		308 (2.6%)	424 (3.6%)	
Native American	745 (0.7%)	874 (0.9%)		118 (1.0%)	131 (1.1%)	
Unknown/ Decline	7057 (7.0%)	7040 (6.9%)		787 (6.7%)	771 (6.6%)	
**US Geographic Region, ** ***n*** **, (%)**			0.03			0.03
South	33,377 (32.7%)	33,796 (33.1%)		3677 (31.4%)	3633 (31.1%)	
Northeast	26,385 (25.9%)	25,294 (24.8%)		2443 (20.9%)	2398 (20.5%)	
Midwest	20,683 (20.3%)	21,847 (21.4%)		2609 (22.3%)	2645 (22.6%)	
West	21,762 (21.3%)	21,270 (20.9%)		2970 (25.4%)	3023 (25.8%)	
**Urban Residence,** *** n*** **, (%)**	75,671 (74.0%)	74,996 (73.4%)	0.02	9046 (77.3%)	9124 (78.0%)	−0.02
**Charlson Comorbidity Index**						
Mean (SD)	1.8 (2.3)	1.7 (2.4)	0.02	0.3 (0.7)	0.3 (0.7)	0.01
Median (IQR)	1 (0–3)	1 (0–3)		0 (0–0)	0 (0–0)	
**Comorbidities of Interest,** *** n*** **, (%)**						
Substance use disorder	43,096 (42.2%)	25,482 (24.9%)	0.37	5762 (49.3%)	2756 (23.6%)	0.55
Mental health conditions of interest (Any)	30,176 (29.5%)	14,980 (14.7%)	0.36	5699 (48.7%)	3208 (27.4%)	0.45
PTSD	17,772 (17.4%)	9213 (9.0%)	0.25	3671 (31.4%)	2163 (18.5%)	0.30
Anxiety	12,622 (12.3%)	5717 (5.6%)	0.24	2452 (21.0%)	1125 (9.6%)	0.32
Suicide ideation	5554 (5.4%)	218 (0.2%)	0.32	1303 (11.1%)	34 (0.3%)	0.48
Suicide attempt	640 (0.6%)	39 (0.0%)	0.10	< 11^a^	< 11 ^a^	–
Hypertension	29,684 (29.0%)	28,142 (27.5%)	0.03	484 (4.1%)	334 (2.9%)	0.07
Hyperlipidemia	27,243 (26.7%)	24,504 (24.0%)	0.06	832 (7.1%)	514 (4.4%)	0.12
Diabetes	21,856 (21.4%)	21,519 (21.1%)	0.01	243 (2.1%)	225 (1.9%)	0.01
Obesity	10,172 (10.0%)	8053 (7.9%)	0.07	791 (6.8%)	496 (4.3%)	0.11
Coronary artery disease	6252 (6.1%)	8489 (8.3%)	−0.08	11 (0.1%)	18 (0.2%)	−0.02
Hepatitis	5405 (5.3%)	2932 (2.9%)	0.12	143 (1.3%)	50 (0.4%)	0.09
Liver disease	4249 (4.2%)	3092 (3.0%)	0.06	148 (1.3%)	83 (0.7%)	0.06
Cancer	2902 (2.8%)	4942 (4.8%)	−0.10	27 (0.2%)	59 (0.5%)	−0.05
Congestive heart failure	2917 (2.9%)	3839 (3.8%)	−0.05	21 (0.2%)	15 (0.1%)	0.01
**PHQ-2 score,** ^**c**^ ***n*** **, (%)**	60,596 (59.3%)	71,602 (70.1)		5961 (51.0%)	7689 (65.7%)	
Mean (SD)	1.1 (1.7)	0.5 (1.2)	0.41	1.8 (2.1)	0.8 (1.5)	0.58
Median (IQR)	0 (0–2)	0 (0)		1 (0–3)	0 (0–1)	

*SCZ* Schizophrenia, *N* Number*, IQR* Interquartile range*, NA* Not applicable*, PHQ-2* Patient Health Questionnaire-2*, PTSD* Posttraumatic stress disorder, *SD* Standard deviation

^a^Counts < 11 are not disclosed to protect patient privacy. ^b^Includes veterans with 35 year of age at index. ^c^ Score ranges from 0 to 6; a score of 3 or higher is considered as the optimal cut-point when using the PHQ-2 to screen for depression

The demographic characteristics for the matched young veteran sub-cohorts (11,699 veterans in each cohort) were similar to the overall schizophrenia cohort, except for the younger age at index (mean and median, 30–31 years) and higher frequency of mental health-related comorbidities (Table 1). Compared with young veterans without schizophrenia, mental health-related comorbidities were more prevalent in young veterans with schizophrenia; these findings were particularly evident for substance use disorder (49.3% of the young veterans with schizophrenia vs. 23.6% of the young veterans without schizophrenia; SMD: 0.55), posttraumatic stress disorder (PTSD; 31.4% vs. 18.5%; SMD: 0.30), and suicidal ideation (11.1% vs. 0.3%; SMD: 0.48).

### Non-healthcare societal outcomes

**Fig. 2:**
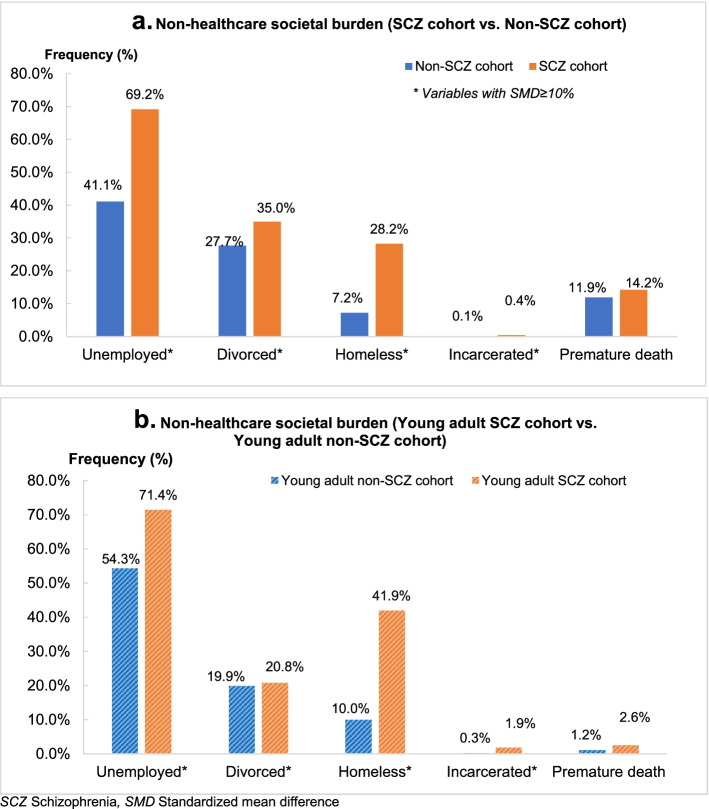
Non-healthcare societal burden

For all societal outcomes, the frequency of events was higher for the schizophrenia cohort compared with the non-schizophrenia cohort (Fig. 2a). Compared with matched veterans without schizophrenia, a greater proportion of the schizophrenia cohort were unemployed (69.2% vs. 41.1%; SMD: 0.81), divorced (35.0% vs. 27.7%; SMD: 0.67), homeless (28.2% vs. 7.2%; SMD: 0.57), or incarcerated (0.4% vs. 0.1%; SMD: 0.47; Fig. 2a).

A similar trend was observed for young veterans with schizophrenia, for whom the frequency of events of interest was greater than matched young veterans without schizophrenia (SMD > 0.1; Fig. 2b). Compared with matched young veterans without schizophrenia, a greater proportion of the young veterans with schizophrenia were unemployed (71.4% vs. 54.3%; SMD: 0.85), divorced (20.8% vs. 19.9%; SMD: 0.46), homeless (41.9% vs. 10.0%; SMD: 0.78), or incarcerated (1.9% vs. 0.3%; SMD: 0.68) (Fig. 2b).

### Odds of unemployment and homelessness

From the adjusted analyses (Table 2), veterans with schizophrenia had 5.4 times (95% CI: 5.29–5.57) greater odds of unemployment and 4.5 times (95% CI: 4.40–4.67) greater odds of homelessness compared with veterans without schizophrenia. Black veterans (OR, 1.2 [95% CI: 1.14–1.21]) and those with a history of substance use disorder (OR, 1.6 [95% CI: 1.56–1.65]) had a greater risk of unemployment, regardless of schizophrenia status. The odds of homelessness were 3.4 times (95% CI: 3.25–3.59) greater for veterans aged 18–34 years compared with veterans 65 years and older and 2.4 times (95% CI: 2.37–2.51) greater for Black versus White veterans, regardless of schizophrenia status. Prior trauma, substance use disorder, and suicidal ideation/attempts were also strong predictors (OR, 2.4–2.8) of homelessness (Table 2).

**Table 2:** Risk of unemployment and homelessness (matched SCZ cohort versus non-SCZ cohort)

	Risk of unemployment	Risk of homelessness
	Adjusted odds ratio (95% CI)	Adjusted odds ratio (95% CI)
**SCZ (vs. non-SCZ cohort)**	**5.43 (5.29−5.57)**	**4.53 (4.40−4.67)**
Age Group (vs. 65 and older)		
18–34	0.75 (0.71−0.78)	3.42 (3.25−3.59)
35–44	0.50 (0.48−0.53)	3.25 (3.08−3.42)
45–54	0.52 (0.50−0.54)	3.25 (3.11−3.39)
55–64	0.75 (0.71−0.78)	2.57 (2.47−2.67)
Sex		
Male (vs. female)	0.93 (0.90−0.97)	1.26 (1.21−1.32)
Race (vs. White)	–	–
Black	1.17 (1.14−1.21)	2.44 (2.37−2.51)
Other	0.97 (0.93−1.01)	1.01 (0.96−1.06)
US Geographic Region (vs. South)		
Midwest	1.03 (1.00−1.07)	1.19 (1.15−1.24)
Northeast	0.99 (0.96−1.03)	1.29 (1.24−1.33)
West	1.09 (1.06−1.13)	2.39 (2.31−2.48)
CCI (continuous)	1.13 (1.12−1.13)	0.97 (0.97−0.98)
Mental Health-Related Comorbidities (vs. none)		
PTSD	1.09 (1.05−1.13)	1.13 (1.09−1.17)
Trauma	1.02 (0.94−1.11)	2.41 (2.23−2.61)
Anxiety	1.01 (0.97−1.06)	1.21 (1.16−1.25)
Substance use disorder	1.60 (1.56−1.65)	2.40 (2.34−2.46)
Suicidal ideation and attempts	0.97 (0.89−1.06)	2.81 (2.65−2.98)

*CCI* Charlson Comorbidity Index*, PTSD* Posttraumatic stress disorder, *SCZ* Schizophrenia, *CI* Confidence interval

Increased odds of homelessness were also observed among the sub-cohort of young veterans with schizophrenia (results not shown); the odds of homelessness were 5.1 times (95% CI: 4.78–5.55) greater in the young veterans with versus without schizophrenia. In this model, young veterans with history of substance use disorder had 2.4 times greater odds of homelessness compared with veterans without history of substance use disorder.

### Premature death

The mean (SD) follow-up time was 4.2 (1.8) years for the schizophrenia cohort and 3.8 (1.9) years for the non-schizophrenia cohort. Of the veterans who died during the observable follow-up (17.0% vs. 19.3%; SMD: − 0.06), the average age at death was 69 years for the schizophrenia cohort and 74 years for the non-schizophrenia cohort (SMD: − 0.42). A total of 83.4% and 61.7% of the veterans who died in the schizophrenia and non-schizophrenia cohorts, respectively, were considered to have died prematurely (SMD: 0.07). The 3 most common causes of premature death among veterans with schizophrenia were cardiovascular disease (15.1%), cancer (14.0%), and chronic lower respiratory disease (5.6%). Substance abuse (2.8%) and intentional self-harm (i.e., suicide; 1.7%) ranked within the top 10 causes of premature death.

## Discussion

Overall, our findings are consistent with the literature and highlight the significant burden of schizophrenia among veterans utilizing VHA services. The study population was predominantly male, White veterans in their 50s–60s; similar to previous studies on US veterans with schizophrenia or schizoaffective disorder [29, 30]. Veterans with schizophrenia in our study demonstrated a greater frequency of mental health comorbidities and substance use disorder compared with the non-schizophrenia cohort (42.2% vs. 24.9% for substance use disorder, 17.4% vs. 9.0% for PTSD, 12.3% vs. 5.6% for anxiety disorder, 5.4% vs. 0.2% for suicide ideation). These findings are consistent and near or within the range of previous studies reported for veterans with schizophrenia (24.6–47.0% for substance use disorder, 22.4–67.0% for PTSD, 13.3–65.0% for anxiety disorder) [2, 31–33] and, as expected, higher than those of the general population (9.9% for substance use disorder, 6.8% for PTSD, 2.7% for anxiety disorder) [34–36]. The frequency of unemployment for veterans with schizophrenia was greater than veterans without schizophrenia (69.2% vs. 41.1%) and the general US population (6.9%) [14]. The frequency of homelessness was also greater for veterans with schizophrenia than those without (28.2% vs. 7.2%) and greater than has been observed in the general population (0.2%) [37].

Although our study did not evaluate costs and causality cannot be confirmed, our findings of societal outcomes may potentially infer substantial associated economic burden. For example, the number of veterans with schizophrenia who experienced homelessness in the present study (*n* = 4903) multiplied by the estimated annual cost of sheltering an unhoused individual ($18,668) [38] yields a cost exceeding $90 million USD per year. In adjusted analyses, the odds of homelessness among veterans with schizophrenia were 4 times higher than those without schizophrenia. We also found that Black race (vs. White), younger age, geographic region, and comorbid mental health conditions were strong predictors of homelessness, suggesting health disparities within the veteran population above and beyond schizophrenia. Causality attribution notwithstanding, our findings shed light on an existing complex interplay between social determinants of health (e.g., housing, socioeconomic status, employment) and health disparities, as previously reported [39, 40], and on the potential association between social determinants of health and societal burden of schizophrenia, further highlighting the importance of targeted, multifactorial interventions for groups that experience health disparities.

In addition, a subset of young veterans with schizophrenia was evaluated in this study because they were expected to be at an early stage of the disease, a time at which patients are at greater risk of relapse due to poor adherence to medications and, hence, have a greater disease burden [18–20]. Our findings confirmed this increased risk and associated outcomes among young veterans. The significant burden of mental illnesses that young veterans with schizophrenia carry was clear, with half having substance use disorder, nearly one-third experiencing PTSD, and more than 10% experiencing suicidal ideation. The frequencies of these conditions were numerically lower in young veterans without schizophrenia, with approximately one-quarter having substance use disorder, approximately one-fifth having PTSD, and less than 1% experiencing suicidal ideation; nonetheless, the prevalence of these conditions remains high, similar to reports from other studies on the younger VHA population (substance misuse in 14–32% of veterans aged 18–34 years [41], PTSD in 20–30% of veterans aged 18–29 years [42, 43], and suicidal ideation in 0.6% of veterans aged 18–25 years) [44]. The majority of young veterans were unemployed, with a greater frequency of unemployment among young veterans with schizophrenia compared to those without schizophrenia (71.4% vs. 54.3%), likely reflecting the findings from previous studies reporting an association between younger age and poorer social functioning [20, 29, 45–47]. Furthermore, more than 40% of young veterans with schizophrenia had evidence of homelessness; this was numerically higher than the overall schizophrenia cohort (28%) and the young non-schizophrenia cohort (10%).

Our study also found a more frequent occurrence of premature death among veterans with schizophrenia, with 14.2% meeting our criteria for premature death compared with 11.9% of veterans without schizophrenia. Furthermore, the mean age of death was 69 years in veterans with schizophrenia, 5 years younger than that for veterans without schizophrenia. This finding is consistent with a large survey of veterans, in which those with schizophrenia died earlier of heart disease than those with no mental health disorder diagnoses (68.6 years vs. 76.5 years) [48], and with a prior meta-analysis of 11 global studies reporting that schizophrenia was associated with a weighted average of 14.5 years of potential life lost [49]. Although our findings on the most common causes of premature death in veterans with schizophrenia were similar to those of the general US population (cardiovascular disease and cancer) [50, 51], our study found that suicide was more prevalent among veterans than in the general US population [50]. Also, substance use disorder was the fourth most common cause of premature death in veterans with schizophrenia; substance abuse is not one of the most common causes of death in the general US population [52].

Our findings emphasize the need for early interventions in patients with schizophrenia, including non-pharmacological and pharmacological interventions aimed at preventing relapse, reducing hospitalization, and managing comorbidities such as mental health and substance use disorder. For example, the literature suggests a positive impact of non-pharmacological interventions such as vocational services along with psychotherapy or housing services on health outcomes [26, 27]; the American Psychiatric Association recognizes this and recommends patients with schizophrenia receive supported employment services [1]. Because a large proportion of patients with schizophrenia struggle with medication adherence, which contributes to disease relapse and poor outcomes [1], treatment strategies should include selection of agents that foster improved adherence. Studies have demonstrated the effectiveness of long-acting injectables (LAI APs) in improving medication adherence and reducing healthcare utilization compared to oral treatment [53, 54]. Notably, within the veteran population, use of an LAI versus an oral atypical antipsychotic was associated with a lower likelihood of homelessness [54]. However, LAIs are underutilized in the real-world setting [1, 55–59]. A recent large study of US veterans using VA health services reported that veterans with psychotic disorder and bipolar disorder who are homeless or unstably housed (HUH) were less likely to be prescribed any antipsychotic treatment compared to non-HUH veterans; use of LAI antipsychotics were also less frequent in HUH veterans [60]. Furthermore, clinical studies report that schizophrenia-related changes in the brain occur within 5 years after the first acute episode, and failure to achieve remission from the initial episode can lead to treatment-refractory schizophrenia, which in turn results in worse outcomes such as homelessness and suicide [61–63]. Thus, identifying schizophrenia early and removing barriers to treatment adherence are both imperative in managing the disease and mitigating the disease burden [20, 45–47].

To our knowledge, our study is the first to evaluate the frequency of non-healthcare societal outcomes in schizophrenia in the US veteran population. Strengths of our study include utilization of the VHA database which provided a large sample size, long follow-up period (mean, 4 years), and access to societal measures not typically available in administrative claims and electronic medical records data. The database also allowed for a more comprehensive view of patient journey and health care receipt; veterans are less likely to seek care for mental health outside of the VHA system due to limited private providers of veteran-focused health services [64]. In addition, mortality is accurately captured in the VHA’s Vital Status File, as it synthesizes data from multiple internal and external sources (e.g., NDI) and is updated daily.

Despite these strengths, there are several limitations of this study. Given that the study population is limited to veterans obtaining health care through the VHA system, the study findings may not be generalizable to the general US population. For example, women in particular were substantially underrepresented. The VHA population has been reported to have particularly high prevalence of mental health conditions, including substance use disorder and PTSD, when compared to the general US population [4]. Moreover, there may be other VA-specific factors such as military screening, combat experience, and coverage for mental health management that make our study population unique. In addition, it is important to note the attrition in the schizophrenia cohort. After the application of inclusion and exclusion criteria, the schizophrenia cohort included 119,343 patients, 17,136 (14%) of whom could not be matched from within the non-schizophrenia cohort. This lack of matching could result in underrepresentation of a subset of the schizophrenia cohort, thereby limiting the generalizability of our findings. Secondly, the societal burden outcomes were captured in a cross-sectional approach using the full study period, with the most recent measure reported, which may potentially underestimate the societal burden over time as the disease progresses. Moreover, only the risks of homelessness and unemployment were further adjusted for variables such as mental health-related comorbidities and substance use disorder in logistic regression models because homelessness and unemployment were known a priori to be predictive of the health outcomes in the schizophrenia population [26, 27]. It is also possible that homelessness and unemployment contribute to the risk of each other, and our study did not investigate this. Future studies assessing the risk of homelessness while controlling for employment status and vice versa, as well as adjusted analyses assessing the risk of other non-healthcare burden measures (divorce and incarceration) may be beneficial.

Furthermore, our study data did not capture costs; therefore, we did not quantify the cost of outcomes we examined. Future studies quantifying the cost of outcomes we measured are also warranted. Lastly, the divorce rate in the schizophrenia cohort may be underestimated because it was not assessed among veterans with schizophrenia who were married; the marriage rate among veterans with schizophrenia was lower than in the non-schizophrenia cohort.

## Conclusion

In this population of patients treated in the VHA system, greater likelihood of adverse non-healthcare societal outcomes were observed among veterans with schizophrenia versus without schizophrenia. Given their elevated risk for unemployment and homelessness, veterans with schizophrenia should be a focus of targeted, multifactorial interventions to reduce disease burden.

## Data Availability

The data that support the findings of this study are available from the Veterans Health Administration, but restrictions apply to the availability of these data, which were used under license for the current study, and so are not publicly available. Data are, however, available from the authors upon contracted agreement and with the permission of Veterans Health Administration and Janssen Scientific Affairs. Please contact the corresponding author, Keiko Wada (keiko.wada@gmail.com), to request the data from this study.

## Abbreviations

CCI: Charlson Comorbidity Index
CI: Confidence interval
ED: Emergency department
HUH: Homeless or unstably housed
LAIs: Long-acting injectables
NDI: National Death Index
OR: Odd ratio
PHQ-2: Patient Health Questionnaire-2
PTSD: Posttraumatic stress disorder
SD: Standard deviation
SMD: Standardized mean difference
US: United States
VHA: Veterans Health Administration

## References

[1] American Psychiatric Association. Practice guideline for the treatment of patients with schizophrenia. Third ed. Washington, DC: American Psychiatric Association; 2020.

[2] Trivedi RB, Post EP, Sun H, Pomerantz A, Saxon AJ, Piette JD (2015). Prevalence, comorbidity, and prognosis of mental health among US veterans. Am J Public Health.

[3] Olfson M, Gerhard T, Huang C, Crystal S, Stroup TS (2015). Premature mortality among adults with schizophrenia in the United States. JAMA Psychiatry.

[4] Veterans Association Health Services Research & Development Serious Mental Illness Treatment Resource and Evaluation Center. Care for Veterans With Psychosis in the Veterans Health Administration, FY09. Ann Arbor, Mich: 11th Annual National Psychosis Registry Report; 2009.

[5] McCarthy JF, Bossarte RM, Katz IR, Thompson C, Kemp J, Hannemann CM, et al. Predictive modeling and concentration of the risk of suicide: implications for preventive interventions in the US Department of Veterans Affairs. Am J Public Health. 2015;105:1935–42. 10.2105/AJPH.2015.302737. 10.2105/AJPH.2015.302737 PMC4539821 26066914

[6] Hunt MG, Cuddeback GS, Bromley E, Bradford DW, Hoff RA (2019). Changing rates of mental health disorders among veterans treated in the VHA during troop drawdown, 2007-2013. Community Ment Health J.

[7] Goren JL, Rose AJ, Smith EG, Ney JP (2016). The business case for expanded clozapine utilization. Psychiatr Serv.

[8] Wu EQ, Birnbaum HG, Shi L, Ball DE, Kessler RC, Moulis M (2005). The economic burden of schizophrenia in the United States in 2002. J Clin Psychiatry..

[9] Cloutier M, Aigbogun MS, Guerin A, Nitulescu R, Ramanakumar AV, Kamat SA (2016). The economic burden of schizophrenia in the United States in 2013. J Clin Psychiatry.

[10] Wyatt RJ, Henter I, Leary MC, Taylor E. An economic evaluation of schizophrenia–1991. Soc Psychiatry Psychiatr Epidemiol. 1995;30:196–205. 10.1007/BF00789054 PMC4300526 7482004

[11] Gunderson JG, Mosher LR (1975). The cost of schizophrenia. Am J Psychiatry.

[12] Desai PR, Lawson KA, Barner JC, Rascati KL (2013). Estimating the direct and indirect costs for community-dwelling patients with schizophrenia. J Pharm Health Serv Res.

[13] Chong HY, Teoh SL, Wu DB, Kotirum S, Chiou CF, Chaiyakunapruk N (2016). Global economic burden of schizophrenia: a systematic review. Neuropsychiatr Dis Treat.

[14] United States Bureau of Labor Statistics (2020). The employment situation-October 2020.

[15] Ramsay CE, Goulding SM, Broussard B, Cristofaro SL, Abedi GR, Compton MT (2011). Prevalence and psychosocial correlates of prior incarcerations in an urban, predominantly African-American sample of hospitalized patients with first-episode psychosis. J Am Acad Psychiatry Law.

[16] Ayano G, Tesfaw G, Shumet S (2019). The prevalence of schizophrenia and other psychotic disorders among homeless people: a systematic review and meta-analysis. BMC Psychiatry..

[17] Rosenheck R, Kasprow W, Frisman L, Liu-Mares W (2003). Cost-effectiveness of supported housing for homeless persons with mental illness. Arch Gen Psychiatry.

[18] Manjelievskaia J, Amos TB, El Khoury AC, Vlahiotis A, Cole A, Juneau P (2018). A comparison of treatment patterns, healthcare resource utilization, and costs among young adult Medicaid beneficiaries with schizophrenia treated with paliperidone palmitate or oral atypical antipsychotics in the US. J Med Econ.

[19] Pilon D, Muser E, Lefebvre P, Kamstra R, Emond B, Joshi K (2017). Adherence, healthcare resource utilization and Medicaid spending associated with once-monthly paliperidone palmitate versus oral atypical antipsychotic treatment among adults recently diagnosed with schizophrenia. BMC Psychiatry.

[20] Immonen J, Jaaskelainen E, Korpela H, Miettunen J (2017). Age at onset and the outcomes of schizophrenia: a systematic review and meta-analysis. Early Interv Psychiatry.

[21] Huang A, Amos T, Joshi K, Wang L, Nash A. Burden of schizophrenia in recently diagnosed adult patients: a commercial payer perspective. New Orleans, Louisiana: 30th Annual Psych Congress; 2017.

[22] Pompili M, Amador XF, Girardi P, Harkavy-Friedman J, Harrow M, Kaplan K (2007). Suicide risk in schizophrenia: learning from the past to change the future. Ann General Psychiatry.

[23] United States Department of Veteran Affairs. VA mental health services public report. Washington DC: United States Department of Veteran Affairs; 2014. Available from: https://www.mentalhealth.va.gov/docs/Mental_Health_Transparency_Report_11-24-14.pdf.

[24] Kroenke K, Spitzer RL, Williams JB (2003). The patient health Questionnaire-2: validity of a two-item depression screener. Med Care.

[25] Centers for Disease Control and Prevention (US). CDC National Health Report highlights. 2014. Available from: https://stacks.cdc.gov/view/cdc/25808.

[26] Bell MD, Zito W, Greig T, Wexler BE (2008). Neurocognitive enhancement therapy with vocational services: work outcomes at two-year follow-up. Schizophr Res.

[27] Sadowski LS, Kee RA, VanderWeele TJ, Buchanan D (2009). Effect of a housing and case management program on emergency department visits and hospitalizations among chronically ill homeless adults: a randomized trial. Jama..

[28] Lambert M, Sanchez P, Bergmans P, Gopal S, Mathews M, Wooller A (2020). Effect of paliperidone palmitate 3-month formulation on goal attainment and disability after 52 weeks' treatment in patients with clinically stable schizophrenia. Neuropsychiatr Dis Treat.

[29] Thorp SR, Sones HM, Glorioso D, Thompson W, Light GA, Golshan S (2012). Older patients with schizophrenia: does military veteran status matter?. Am J Geriatr Psychiatry.

[30] Kreyenbuhl JA, Valenstein M, McCarthy JF, Ganoczy D, Blow FC (2007). Long-term antipsychotic polypharmacy in the VA health system: patient characteristics and treatment patterns. Psychiatr Serv.

[31] Buckley PF, Miller BJ, Lehrer DS, Castle DJ (2009). Psychiatric comorbidities and schizophrenia. Schizophr Bull.

[32] Muller JE, Koen L, Soraya S, Emsley RA, Stein DJ (2004). Anxiety disorders and schizophrenia. Curr Psychiatry Rep..

[33] Tsai J, Rosenheck RA (2013). Psychiatric comorbidity among adults with schizophrenia: a latent class analysis. Psychiatry Res.

[34] Kessler RC, Berglund P, Demler O, Jin R, Merikangas KR, Walters EE (2005). Lifetime prevalence and age-of-onset distributions of DSM-IV disorders in the National Comorbidity Survey Replication. Arch Gen Psychiatry.

[35] National Institute of Mental Health. Generalized anxiety disorder 2017. Available from: https://www.nimh.nih.gov/health/statistics/generalized-anxietydisorder.shtml#:~:text=An%20estimated%202.7%25%20of%20U.S.,than%20for%20males%20(1.9%25).

[36] Grant BF, Saha TD, Ruan WJ, Goldstein RB, Chou SP, Jung J (2016). Epidemiology of DSM-5 drug use disorder: results from the National Epidemiologic Survey on alcohol and related conditions-III. JAMA Psychiatry..

[37] The Council of Economic Advisers. The state of homelessness in America. 2019. Available from: https://www.whitehouse.gov/wp-content/uploads/2019/09/The-State-of-Homelessness-in-America.pdf.

[38] US Department of Housing and Urban Development. Cost associated with first-time homelessness for families and individuals. 2010. Available from: https://www.huduser.gov/publications/pdf/Costs_Homeless.pdf.

[39] Alegria M, NeMoyer A, Falgas Bague I, Wang Y, Alvarez K (2018). Social determinants of mental health: where we are and where we need to go. Curr Psychiatry Rep.

[40] World Health Organization and Calouste Gulbenkian Foundation (2014). Social determinants of mental health.

[41] Kaplan MS, McFarland BH, Huguet N, Valenstein M (2012). Suicide risk and precipitating circumstances among young, middle-aged, and older male veterans. Am J Public Health.

[42] Lee L (2019). PTSD and aging. PTSD Research Quarterly.

[43] Tanielian T, Jaycox LH, Schell TL, Marshall GN, Burnam MA, Eibner C, et al. Invisible wounds. mental health and cognitive care needs of America's returning veterans: RAND Corporation; 2008.

[44] Logan J, Bohnert A, Spies E, Jannausch M (2016). Suicidal ideation among young Afghanistan/Iraq war veterans and civilians: individual, social, and environmental risk factors and perception of unmet mental healthcare needs, United States, 2013. Psychiatry Res.

[45] Hui CL, Li AW, Leung CM, Chang WC, Chan SK, Lee EH (2014). Comparing illness presentation, treatment and functioning between patients with adolescent- and adult-onset psychosis. Psychiatry Res.

[46] Miyamoto S, Wolfgang FW (2017). The use of long-acting injectable antipsychotics in schizophrenia. Curr Treat Options Psychiatry.

[47] Pencer A, Addington J, Addington D (2005). Outcome of a first episode of psychosis in adolescence: a 2-year follow-up. Psychiatry Res.

[48] Kilbourne AM, Morden NE, Austin K, Ilgen M, McCarthy JF, Dalack G (2009). Excess heart-disease-related mortality in a national study of patients with mental disorders: identifying modifiable risk factors. Gen Hosp Psychiatry.

[49] Galletly CA (2017). Premature death in schizophrenia: bridging the gap. Lancet Psychiatry.

[50] Weiner J, Richmond TS, Conigliaro J, Wiebe DJ (2011). Military veteran mortality following a survived suicide attempt. BMC Public Health.

[51] Brown S, Inskip H, Barraclough B (2000). Causes of the excess mortality of schizophrenia. Br J Psychiatry.

[52] Forehand JA, Peltzman T, Westgate CL, Riblet NB, Watts BV, Shiner B (2019). Causes of excess mortality in veterans treated for posttraumatic stress disorder. Am J Prev Med.

[53] Kelly DL, Wehring HJ, Vyas G (2012). Current status of clozapine in the United States. Shanghai Arch Psychiatry.

[54] Young-Xu Y, Duh MS, Muser E, DerSarkissian M, Faust E, Kageleiry A (2016). Impact of paliperidone palmitate versus oral atypical antipsychotics on health care resource use and costs in veterans with schizophrenia. J Clin Psychiatry..

[55] Correll CU, Citrome L, Haddad PM, Lauriello J, Olfson M, Calloway SM (2016). The use of long-acting injectable antipsychotics in schizophrenia: evaluating the evidence. J Clin Psychiatry..

[56] Heres S, Reichhart T, Hamann J, Mendel R, Leucht S, Kissling W (2011). Psychiatrists' attitude to antipsychotic depot treatment in patients with first-episode schizophrenia. Eur Psychiatry.

[57] Kim B, Lee SH, Yang YK, Park JI, Chung YC (2012). Long-acting injectable antipsychotics for first-episode schizophrenia: the pros and cons. Schizophr Res Treatment.

[58] Patel MX, Haddad PM, Chaudhry IB, McLoughlin S, Husain N, David AS (2010). Psychiatrists' use, knowledge and attitudes to first- and second-generation antipsychotic long-acting injections: comparisons over 5 years. J Psychopharmacol.

[59] Kane JM, McEvoy JP, Correll CU, Llorca PM (2021). Controversies surrounding the use of long-acting injectable antipsychotic medications for the treatment of patients with schizophrenia. CNS Drugs.

[60] Tsai J, Szymkowiak D, Radhakrishnan R. Antipsychotic medication prescriptions for homeless and unstably housed veterans in the veterans affairs health care system. J Clin Psychiatry. 2020;82:20m13372. 10.4088/JCP.20m13372. 10.4088/JCP.20m13372 33296148

[61] Green AI, Canuso CM, Brenner MJ, Wojcik JD (2003). Detection and management of comorbidity in patients with schizophrenia. Psychiatr Clin North Am.

[62] Lehman AF, Lieberman JA, Dixon LB, McGlashan TH, Miller AL, Perkins DO (2004). Practice guideline for the treatment of patients with schizophrenia, second edition. Am J Psychiatry.

[63] Caspi A, Davidson M, Tamminga CA (2004). Treatment-refractory schizophrenia. Dialogues Clin Neurosci.

[64] National Alliance on Mental Illness. Protecting veterans' access to mental health care. 2020. Available from: https://www.nami.org/Advocacy/Policy-Priorities/Improve-Care/Protecting-Veterans-Access-to-Mental-Health-Care.

